# Validation of the Spanish language version of the control of allergic rhinitis and asthma test

**DOI:** 10.1038/s41533-022-00313-8

**Published:** 2022-10-29

**Authors:** Quijano Diana, Ali Abraham, Arevalo Yaicith, Orejuela Peter, Trujillo Juan

**Affiliations:** 1 Otorhinolaringology Department, Fundación Santa Fe de Bogotá, Bogotá, Colombia; 2 Pneumology Department Fundación Neumológica Colombiana, Bogotá, Colombia; 3 Allergology Department, Fundación Neumológica Colombiana, Bogotá, Colombia; 4 Statistical Hospital Universitario San Ignacio, Bogotá, Colombia; 5 ENT Resident, Hospital Militar Central, Universidad Militar Nueva Granada, Bogotá, Colombia

**Keywords:** Respiratory signs and symptoms, Asthma

## Abstract

Allergic rhinitis and asthma are common diseases that frequently coexist, referred to as unified airway disease. There is currently no validated scale in Spanish, which allows simultaneous evaluation of both conditions. A translation from Portuguese to Spanish was therefore performed. It was administered to 120 patients aged between 18 and 70 years whose native language was Spanish and presented a diagnosis of allergic rhinitis and asthma. The reliability, validity and sensitivity to instrument change validations were carried out, as well as the values of minimally relevant clinical differences. Reliability was evaluated using Cronbach´s alpha test on CARAT-global: 0.83 [IC 95% 0.79–0.88]; test and retest evaluation was done with Pearson´s correlation coefficient: 0.6 [IC 95% 0.32–0.77] and the standard error of measurement 3.5 (*p* < 0.005). A confirmatory factor analysis was performed corroborating two factors. Correlation coefficients were not high in the longitudinal validation. Concurrent validity showed an acceptable correlation between CARAT10 asthma ACQ5 and low between allergic rhinitis-VAS. There was a milestone of the controlled disease in the discriminant validity of CARAT10 rhinitis ≥ 8 mean an adequate control, CARAT10-asthma > 16 In this case, CARAT10-asthma value < 16 are interpreted as an inadequate or partial control and values ≥ 16 mean an adequate control and CARAT10-global ≥ 18, patients evaluated with CARAT10 with a result ≥ 18, which would be a patient with both conditions controlled. The minimally relevant clinically important average difference found in the CARAT10 scale was 3.25 (SD 3.77). The CARAT10 scale in Spanish is a standardised, reliable and valid evaluation method on patients with unified airway disease.

## Introduction

Allergic rhinitis and asthma are common diseases, which frequently occur together and are known as a unified airway disease. Epidemiological studies have shown that the prevalence of allergic rhinitis symptoms in asthmatics is close to 80% and that of asthma in patients with rhinitis is between 10% and 40%; in both cases being above that of the general population^1–4^.

These pathologies have great socioeconomic impact on patients and health administrators due to high direct and indirect costs, making it necessary to have an adequate control of the disease and an integral management between otolaryngologists, allergists and pulmonologists.

**Fig. 1:**
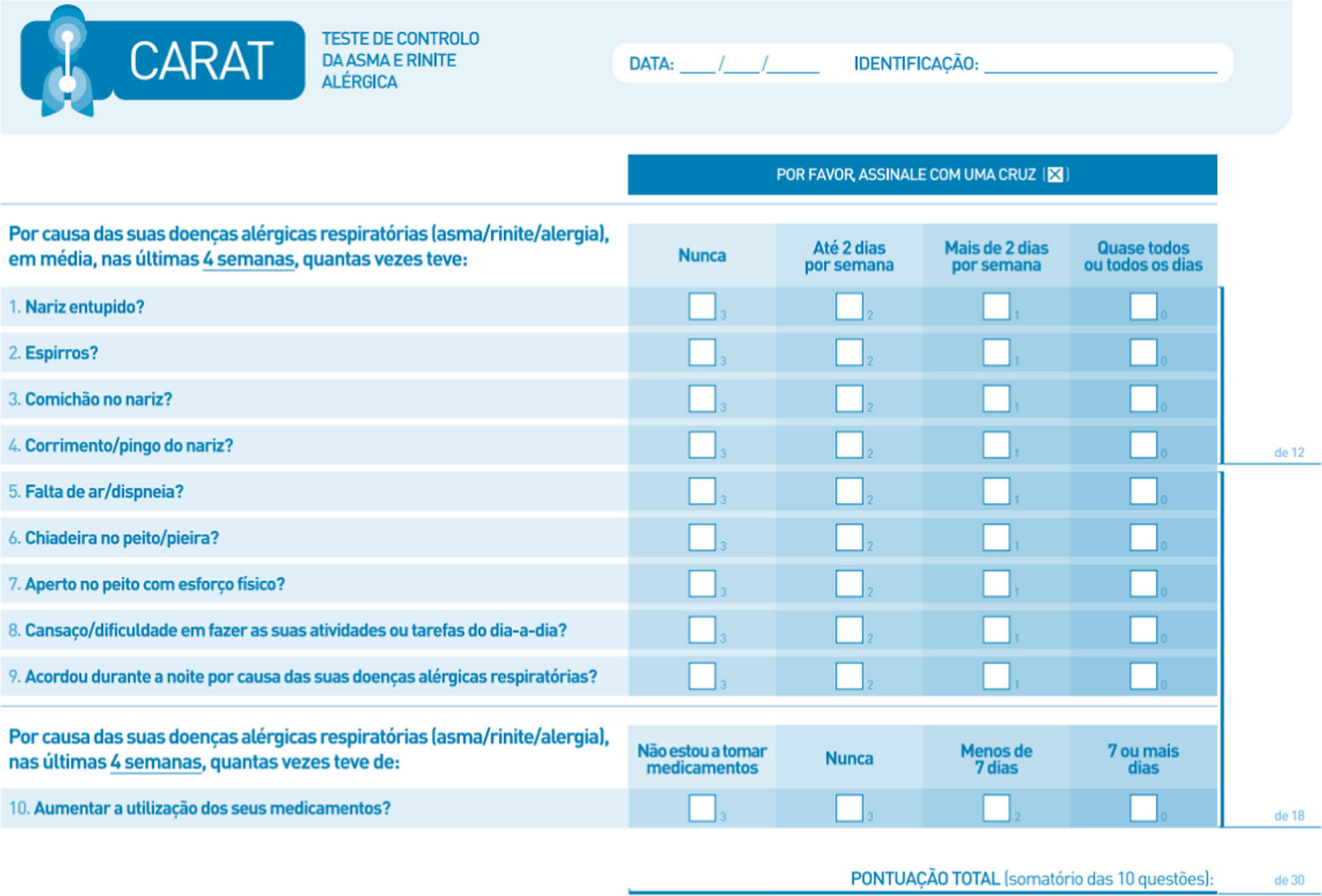
CARAT scale in original Portuguese; ref. ^23^.

The first questionnaire for the simultaneous evaluation and identification of changes in the treatment scheme is the scale of Control of Allergic Rhinitis and Asthma (CARAT10)^5^. Previous validations carried out in Portuguese and German reported the instrument´s adequate reliability and validity^6,7^, for it assesses upper and lower respiratory tract symptoms, interference with sleep, activity limitations and the requirement to increase medication in a period of 4 weeks (Fig. 1).

Participants in the validation studies in the mentioned languages were adults between 18 and 70 years of age with a diagnosis of allergic rhinitis, asthma and at least 6 months of clinical follow-up^6,7^.

Currently, there is no validated scale in Spanish that allows to evaluate said pathologies in a standardised manner and it is necessary in order to valuate this population and facilitate therapeutic decisions. The objective of the present study was to determine the clinimetric properties in Spanish of the scale and compare it with the performance it had in Portuguese.

## Methods

### Study design and procedure

**Fig. 2:**
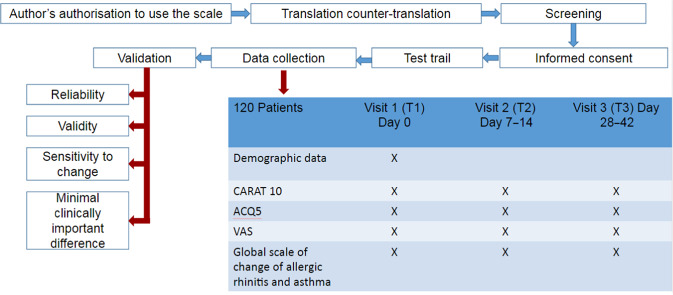
Algorithm to determine the clinimetric characteristics of the CARAT10 scale. VAS visual analogue scale, ACQ5 asthma control questionnaire.

A validation study was performed in order to determine the clinimetric characteristics of the CARAT10 scale whose algorithm is shown in Fig. 2. The original author authorised its use in Spanish and assessment by the authors of the present research^7^. A translation and counter-translation of the scale was applied and a pilot trial with ten patients who later were included in the study.

Patients had three visits: T1 or basal visit, corresponding to the first day in which the scales were applied; T2, between one and 2 weeks; T3, between 4 and 6 weeks after T1. Demographic data and classification of allergic rhinitis and asthma were evaluated in T1. CARAT10, ACQ5, upper, lower and global Analogue-Visual Scale (VAS) were evaluated in T1 and T3. The subjective global change scale was measured during T2 and T3 and the variables were registered in a format developed for the study. Data confidentiality was assured with coding, filed in an Excel® database and exported to the software from this electronic registry for statistical analysis. The study was evaluated and approved by two ethics committees to which the authors belong (Fundación Santa Fé de Bogotá and Fundación Neumológica Colombiana). Written informed consent was obtained from all participants.

### Participants

Patients had ages between 18 and 70 years with a diagnosis of allergic rhinitis, asthma, at least 6 months of clinical follow-up and native Spanish speakers; those who consulted the Colombian Neumology Foundation, which is a third level institute. The diagnosis for allergic rhinitis was determined by clinical evaluation of cardinal symptoms such as nasal obstruction, aqueous rhinorrhea, sneezing, nasal pruritus and physical examination symptoms as turbinate hypertrophy with pale mucous membranes and aqueous rhinorrhea.

The diagnosis for asthma was performed with symptoms and clinical signs of sibilance, dyspnea, coughing and thoracic oppression (denominated guide symptoms) and confirmed by a spirometry test or flow–volume curve. A negative was confirmed by a bronchoconstriction test with exercise or methacholine^8–10^. Patients with cognitive alterations, sensory alterations, and illiterate with conditions that hinder understanding and answering questions were excluded.

### Sample size

The sample size for a valid concurrent criterion was calculated with the following parameters: a sample of at least 68 patients in order to achieve a statistical power of 80%; detection of differences of 0.3 between a nil hypothesis—which means there is no difference—with a correlation of 0.2, an alternate hypothesis or a difference between the CARAT10 in Spanish and the ACQ5 and VAS scales with a correlation of 0.5 and two-tailed hypothesis with a significance of 0.05.

As per recommendations of COSMIN, the test–retest was administered to patients who did not changed their condition in the clinical evaluation during sampling. The CARAT10 scores applied on at least ten patients were taken into account during the T1 (beginning), T2 (2 weeks) and T3 (4 weeks).

A sample of 104 patients was collected for internal consistency with a statistical power of 80%, difference in the Cronbach´s alpha coefficient, nil hypothesis, there wasn’t a good relation between the items in the CARAT10 scale in Spanish of 0.7 and alternate hypothesis, there was a good correlation between the CARAT scale when results were equal or greater than using a two-tail test with a significance of 0.05.

Minimally important clinical differences were considered in order to evaluate sensitivity reported in 3.5 with SD of 2.87^11^ A sample of at least ten patients subjected to repeated scale measurements was required.

According to MacCallum and Widaman, sample sizes of 100 or more persons are proposed for factor analysis and the recommendation is also applicable for confirmatory factor analysis^12,13^.

A sample of ten patients was used per studied item because the scale has that number of features. The minimum sample were 100 patients who completed the required visits^14–16^ and it was determined to complete the sample with 120 patients^6^.

### Reporting summary

Further information on research design is available in the Nature Research Reporting Summary linked to this article.

## Results assessment

CARAT10 comprises ten questions and it is divided into three groups. The first four are for assessing symptoms of allergic rhinitis, the next five are for evaluating asthma symptoms and the last item is related to the increment or not of medication in the previous 4 weeks. It allows an independent evaluation of the upper airway (rhinitis), lower airway (asthma) and unified airway (rhinitis and asthma)^7^. Answer options are designed in a Likert-type scale where the first nine options range from 0 = total control absence, to 3 = total control. Medication answers have options from 0 point to 3 points, where 3 = never, 2 = less than seven days and 0 = more than seven days. Final score has a range of 0 to 30 with 0 being minimal control and 30 total control^7^.

Asthma was assessed using the control scale ACQ5 (Asthma Control Questionnaire ACQ5), which consists of five questions scoring from 0 to 6. The total is calculated by the sum of each item and dividing by five. The range is: less or equal to 0.75 is an adequate control, from 0.75 to 1.50 it is partially controlled and more than 1.50 there is an inadequate control^11^. The scale has been validated in Spanish and it is a valid and reliable instrument^17^.

The Visual Analogue Scale (VAS) was used to assess allergic rhinitis, which measures patient symptoms within a range from 0 mm to 100 mm; 0 mm corresponds to absence of discomfort, 100 mm is intolerable discomfort and the patient must mark with an X the according level. The scale is simple, quantitative and has been widely used for said evaluation and effectiveness of treatments. It was established that 23 mm corresponded to a minimal clinically important difference^18^.

The Global Rating of Change Score is a scale with 15 points used for subjective monitoring of patients´ development of asthma symptoms and allergic rhinitis compared to a previous condition. The scoring interval ranges from −7, which is extremely worse, 0 when there is no change and +7, which is extremely better. The scale was used as an instrument to establish the slightest difference a patient identifies as important, which is the minimal clinically important difference^19^.

### Statistical analysis

The validation to Spanish of the CARAT10 was based on reliability, validity and the instrument´s sensitivity to change. The minimal clinically important difference was calculated. Reliability was established by the internal consistency with Cronbach’s alpha, McDonald´s omega, reliability test–retest and measurement error. Evaluation of measurement error or stability was performed with test–retest after 4 weeks and 6 weeks with Spearman´s correlation and intraclass correlation coefficient. It was applied to patients who did not present a change of condition in their clinical evaluation and Bland Altman graphics were used for concordance evaluation.

Validity was confirmed using factor analysis, longitudinal validity, discriminant validity and concurrent criterion validity. Structural equation analysis methods were used to determine sample adjustment quality of the model in factor analysis because of a previous report of a two-factor structure^7,11^. The statistical *X*^2^/degrees of freedom, Comparative Fit Index (CFI), Tucker-Lewis Index (TLI) and the root mean square error of approximation (RMSEA) were used for quality adjustment estimates. Longitudinal validity was evaluated with correlation coefficients of score differences of repeated measurements between CARAT10, ACQ and analogue-visual scales during T1 and T2. A sample of at least 68 patients was used for this component.

For the discriminant validity based on established ranges for the analogue-visual and ACT evaluations were performed between groups using *t*-tests with values of 0.05 and two-tail hypothesis tests. Additionally, with these ranges, ROC curve analyses were applied (categories generated for ranges were used as gold standards for said curve analysis). These analyses were performed on all assessed patients during T1.

The criterion validity was established by means of Spearman´s correlation, in order to evaluate it between CARAT, ACQ5 and the Analogue-Visual Scale for rhinitis, asthma and global symptoms.

An anchoring method was used in order to determine the minimal clinically important difference by means of change approximation in the intra-patient score^20,21^. It was implemented the same way as^11^ with a Global Rating of Change Score scale (GRC). Patients were divided into four categories based on global score change as follows: no difference (−1, 0. 1), minimal difference (−3, −2, 2, 3), moderate difference (−5, −4, 4, 5) and 7 major difference (−7, −6, 6, 7). CARAT average score in the minimal difference category was used to estimate the MCID.

## Results and discussion

Patient selection was done between March 2020 and January 2021, composed of 120 patients who completed the three evaluations, where 99 (82.5%) were females, 21 (17.5%) males and the average age was 37.7 years. 39% had intermittent allergic rhinitis and 48% moderate asthma; more than half of the latter had access to adequate pathology control (Table 1).

**Table 1:** Population characteristics.

Total of patients	Number : 120
Gender	
Female	99 (82.5%)
Male	21 (17.5%)
Age	
Average	37.3 (SD 13.7)
Median	35.0 [18.0, 70.0]
Categories	
18–34	55 (45.8%)
34–44	28 (23.3%)
44–54	19 (15.8%)
54–70	18 (15.0%)
Allergic rhinitis	
Intermittent	47.0 (39.2%)
Persistent	73.0 (60.8%)
Slight	62.0 (51.7%)
Moderate to severe	58.0 (48.3%)
Asthma ACQ5	
Partially controlled	31.0 (25.8%)
Adequate asthma control	58.0 (48.3%)
Inadequate asthma control	31.0 (25.8%)

*SD* standard deviation.

The scale´s original author provided an official translation to Spanish by official translators, native Spanish speakers and bilingual. The researchers carried out a semantic evaluation of the Spanish version and after some changes, an official translator made the counter-translation from Spanish to Portuguese with review and approval by the original author.

**Fig. 3:**
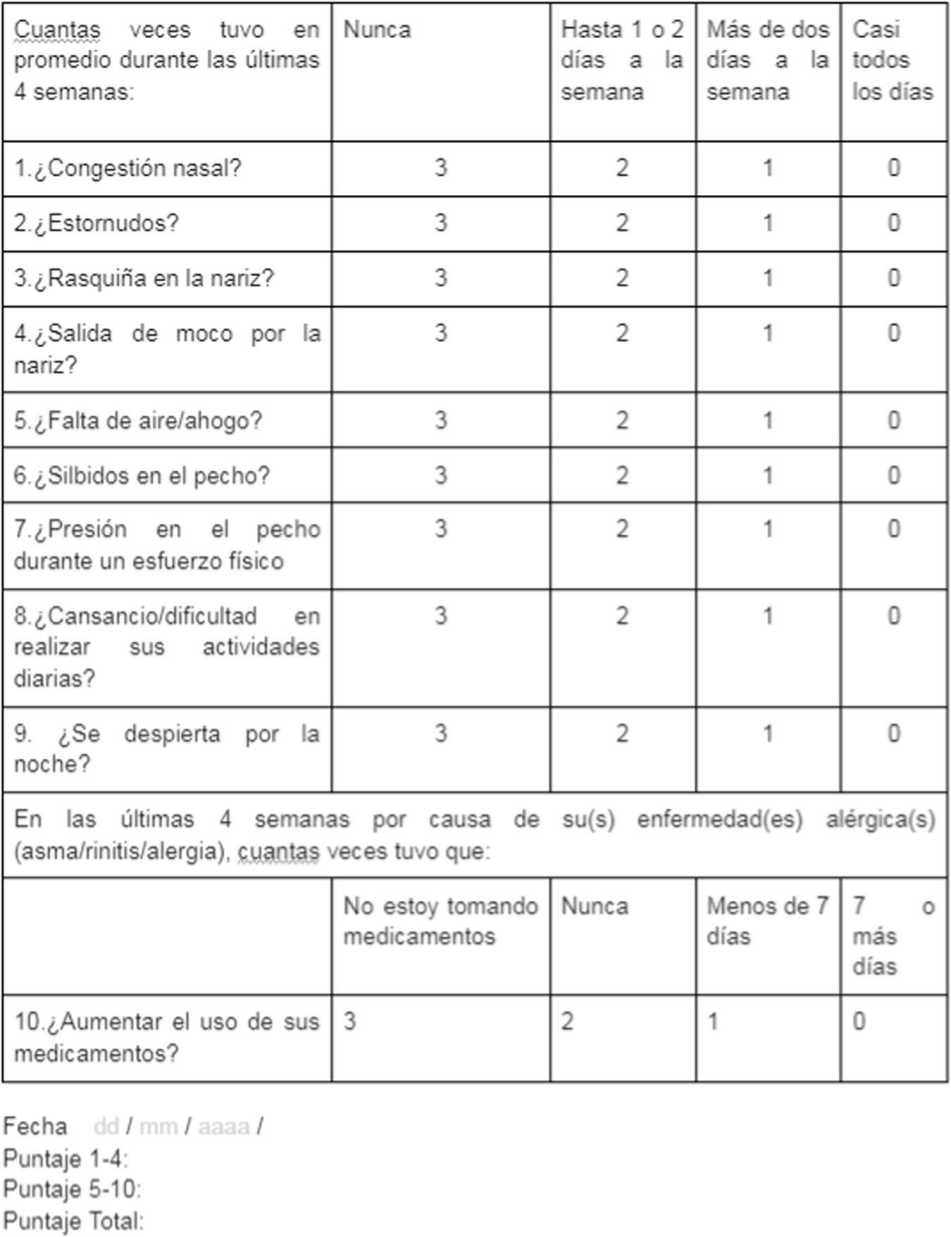
Scale for asthma and allergic rhinitis control CARAT-self-administered in Spanish.

The CARAT10 scale was self-administered in Spanish to ten patients (Fig. 3). Afterwards, an evaluation of each item was carried out as per proposed guidelines by the FACIT organisation for trans-cultural adaptations^22^. The average time per test were 8.7 min (DS 3.16) and all questions were verified to have an answer. Patients found the language to be clear, easy to understand and they were included in the total sample.

Based on recommendations by the Consensus-Based Standards for the Selection of Health Measurements (COSMIN), the validation of a scale was performed with a reliability evaluation, validity and sensitivity to change of the instrument, as well as establishing the values for the minimal clinically important difference.

The reliability was assessed in two ways: internal consistency and measurement error.

Internal consistency evaluated by Cronbach´s alpha was of 0.82 for allergic rhinitis during T1(IC 95% 0.77–0.88), asthma of 0.82 (IC 95% 0.77–0.87) and global of 0.83 (IC 95% 0.79–0.88). There were no statistically significant differences between T2 and T3, obtaining a good level among visit sequences. This was similar to that yielded in the original validation, which was 0.85, and the German validation, which was 0.87^7,11^. The Spanish CARAT (total and domains) showed satisfactory internal consistency, which was comparable to Portuguese and German validation. A Spearman´s alpha correlation, Kendall and polychoric were carried out without significant differences. McDonald´s omega was done in T1 0.88 (IC 95% 0.87–0.94), which was considered a good level without variation of the other visit sequences.

Reliability test–retest was determined by means of the intraclass correlation coefficient and the Bland Altman graphs whose concordance limits were 10.32 and 10.76 in 46 patients with a critical difference of 10.54 between T1 and T3.

Additionally, a Pearson correlation test was applied with a result of 0.6 (IC 95% 0.32–0.7) between T1 and T2 on 35 patients; 0.73 (IC 95% 0.55–0.84) on 46 patients between T2 and T3 obtaining an accurate measurement with moderate correlation after 4 weeks with ample confidence intervals.

The standard error of measurement (SEM) was 3.5 with *p* < 0.005 calculated on 28 participants and it was statistically significant similar to that found in the Van der Leeuw study, which was 2.85^11^. The minimal detectable difference was 9.6, which was high for the scale´s total score.

Validity was obtained with factorial analysis, longitudinal validity, discriminant validity and concurrent criterion validity. The number of assessed patients must be taken into account and the scales compared with CARAT for results analysis. Not all of the 120 patients were involved in the total analysis, which leads to more ample confidence intervals.

Evaluation of asthma was carried out comparing it with the ACQ5. This scale presents two different considerations from CARAT10, which may affect correlation and validation. The first one are three levels of asthma classification: adequate control, partially controlled and inadequate control, while the CARAT10 only has two: controlled and not controlled. The second is the ACQ5’s evaluation is transverse and the patient’s condition is asked at the time of evaluation; CARAT10 has a longitudinal assessment estimating the clinic only in the previous 4 weeks. However, ACQ5 is the scale validate in Spanish are most widely used by pulmonologists.

CARAT10 was compared with VAS regarding allergic rhinitis having 60/100 as cut-point, where above 60 is not controlled and below 60 is controlled. The VAS, as previously explained with ACQ5 (evaluates symptoms at the time of consultation) presents differences in the temporality of the evaluation. Structural equation analysis were used for confirmatory factorial analysis, confirming the structure of two factors as in the original article^7^

Correlation coefficients were used for longitudinal validation between score differences and repeated measurements of CARAT10, ACQ5 and VAS during T1-T2, T2-T3 and T1-T3 in 120 patients. The coefficients were not high; the temporality evaluation between scales was different, which may explain the outcome (Table 2).

**Table 2:** Longitudinal validity. Correlation coefficients.

	Period 1 T1–T2	Period 2 T2–T3	Period 3 T1–T3
CARAT10 total—ACQ5	0.36	0.25	0.30
CARAT10 total—sum VAS allergic rhinitis, asthma	0.37	0.19	0.41
CARAT10 rhinitis—VAS allergic rhinitis	0.22	0.28	0.37
CARAT10 asthma—VAS asthma	0.27	0.37	0.32
CARAT10 asthma—ACQ5	0.42	0.36	0.42

**Fig. 4:**
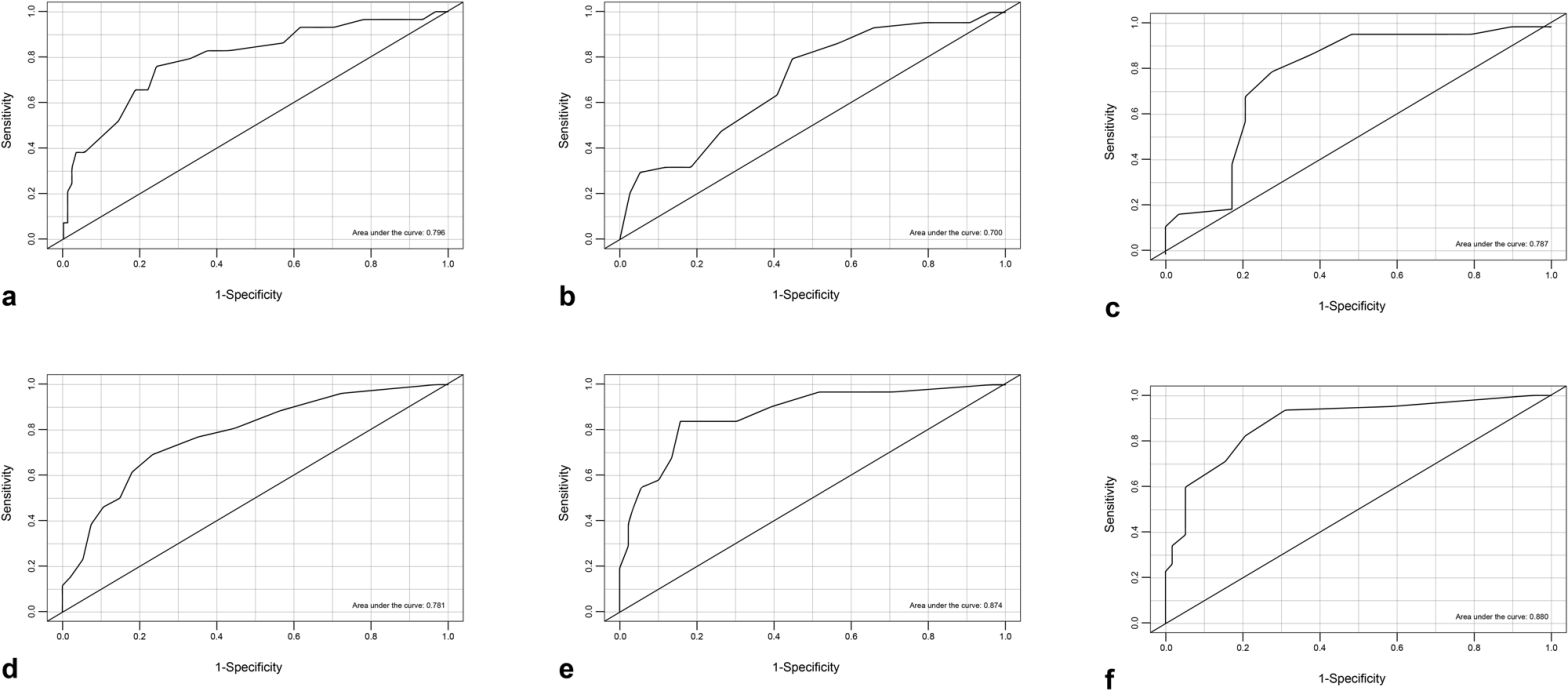
Graph with the operative characteristics of the trial. **a** VAS allergic rhinitis + Asthma vs. CARAT10. **b** VAS (60) allergic rhinitis vs. CARAT10 allergic rhinitis. **c** VAS (30) allergic rhinitis vs. CARAT10 allergic rhinitis. **d** VAS (60) Asthma vs. CARAT10 asthma. **e** ACQ5 vs. CARAT10 asthma. **f** Graphic curve ROC. ACQ5 vs. CARAT10 asthma.

It must be taken into account for discriminant validity that CARAT10 assesses the upper airway (rhinitis), lower airway (asthma) and unified airway (rhinitis and asthma). Results are presented categorised in three groups compared with cut points for VAS and ACQ5. Comparisons were performed on all evaluated patients during T1 using *t*-tests with 0.05 significance levels and two-tail hypothesis trials. ROC curves (operative characteristics of the trial) were carried out on the cut points (Fig 4) and assessed with the Wilcoxon rank test, which was statistically significant. Those results are in Table 3; cut points can be visualised on the CARAT10 in Spanish with its respective sensitivity, specificity and positive and negative predictive values.

**Table 3:** Discriminant validity. Cut points and operative characteristics.

	Cut-point	Cut-point CARAT	Sensitivity (%)	Specificity (%)	PPV	NPV
VAS asthma + allergic rhinitis vs. CARAT10	VAS > 60 Not controllled, VAS < equal to 60 controlled	18: > or equal to 18 controlle, < 18 not controlled	76	76	50	91
VAS allergic rhinitis vs. CARAT10 rhinitis	VAS > 60 Not controlled, VAS < equal to 60 controlled	7: > or equal to 7 controlled, < 7 not controlled	79	55	50	82
VAS allergic rhinitis vs. CARAT10 rhinitis	VAS > 30 partially controlled or not controlled, VAS > or equal to 30 controlled	8:> or equal to 8 controlled, < 8 not controlled	80	72	90	53
VAS asthma vs. CARAT10 asthma	VAS > 60 not controlled, VAS < equal to 60 controlled	13:> or equal to 13 controlled, < 13 not controlled	69	76	45	90
ACQ5 > 1,50 vs. CARAT10	ACQ < or equal to 0.75: adequate asthma control, from 0.75 to 1.50: partially controlled asthma, > 1.50: inadequate asthma control	13:> or equal to 13 adequate asthma control or partially controlled, < 13 inadequate asthma control	83	84	65	93
ACQ5 < or equal to 0,75 vs. CARAT10 asthma	ACQ < or equal to 0.75: adequate asthma control From 0.75 to 1.50: partially controlled asthma > 1.50: inadequate asthma control	16: > or equal to 16 adequate asthma control < 16, inadequate asthma control or partially controlled	93	68	76	90

The VAS-global analysis (asthma+allergic rhinitis) vs. CARTA10-global yielded a cut-point of 18. In this case, a value of CARAT10 < 18 meant that both pathologies were not controlled; a negative predictive value (NPV) of 91% can be interpreted that out of 100 patients evaluated with CARAT10 with a result ≥ 18 (which would be a patient with both conditions controlled), 91 would be controlled upon comparing VAS < 60 (Table 3). Analysing the positive predictive value (PPV) of 50%, among 100 patients with a CARAT10-global < 18 (both conditions not controlled), in reality 50 would not have both of them controlled compared with the VAS-global > 60.

In the upper airway category (CARAT10-rhinitis) vs. VAS-rhinitis the analysis was with two cut points different in the VAS: 30 and 60. These were 8 and 7, respectively, and upon raising it one point (from 7 to 8), it presented a better performance with a positive predictive value of 90%. With a cut-point < 7 the positive predictive value diminishes to 50%, confirming that the best cut-point was 8.

The lower airway evaluation comprised two analyses, comparing CARAT10-asthma vs. VAS-asthma and CARAT10-asthma vs. ACQ5. The CARAT10-asthma vs. VAS-asthma evaluation had a cut-point in the VAS > 60, understood as an uncontrolled condition. In the CARAT10-asthma corresponds to a cut-point < 13 and a high negative predictive value of 90%.

The comparison of CARAT10-asthma vs. ACQ5 was with two analyses of the three categories of the latter. The first one compared a cut-point ACQ5 > 1.5 meaning patients with an inadequate control of asthma, equivalent to a cut-point of 13 in the CARAT10-asthma and a negative predictive value of 93%. In this case, CARAT10-asthma < 13 means an inadequate control and values ≥ 13 are understood as partial or total control.

The comparison of a cut-point ACQ5 > 0.75 represents patients with partial or inadequate asthma control. An equivalent point of 16 was found in CARAT10 with a negative predictive value of 90%. In this case, CARAT10-asthma values < 16 are interpreted as an inadequate or partial control and values ≥ 16 mean an adequate control. It is more useful to clearly differentiate patients with an adequate asthma control so a CARAT10-asthma cut-point of 16 in clinical practice is suggested.

In the concurrent criterion validity, the correlation between CARAT10, ACQ5 and VAS was measured in T1 and all 120 patients, applying the scales simultaneously (Table 4). Correlation of CARAT10-asthma and ACQ5 was acceptable and allergic rhinitis-VAS was low. The correlation between CARAT10-global with VAS-rhinitis+asthma was 0.63, existing a concurrent criterion validity with similar results in T2 and T3.

**Table 4:** Validity of concurrent criteria T1.

	ACQ5	VAS asthma	VAS allergic rhinitis	VAS allergic rhinitis + Asthma
CARAT10 total	−0.66	−0.56	−0.53	−0.63
CARAT10 allergic rhinitis	−0.36	−0.29	−0.49	−0.45
CARAT10 asthma	−0.74	−0.65	−0.42	−0.62

The minimal clinically important difference was determined using the distribution anchor method^21^ (−1, 0, +1: no difference; −3, −2, 2, 3: minimal difference; −5, −4, 4, 5: moderate difference; −7, −6, 6, 7: large difference.) The average minimal clinically important difference with CARAT10 was 3.25 (SD 3.77) similar to the German validation of 3.5^11^.

One of the strengths of this study was its design based on the COSMIN guides for validation of scales. The calculated sample size was 120 patients, had a diagnosis of asthma and allergic rhinitis, all of whom completed the study. The results of validation are only applicable to patients diagnosed with both pathologies.

This is the first CARAT validation study that established the cutoff points to determine if rhinitis, asthma and both pathologies are controlled. It is a useful measurement tool in daily practice in patients with asthma and allergic rhinitis.

CARAT Spanish scale validation has been carried out with an adequate and rigorous clinical an statistical design, following COSMIN guides, the finding found have internal validity and can be extrapolated to the Spanish speaking population.

In conclusion, CARAT10 scale in Spanish presents a high internal consistency, good discriminant and concurrent validity. A standardised measurement method allows simultaneous evaluation of asthma control and allergic rhinitis.

Important results for clinical practice were established in this study such as the minimal clinically significant difference and cut points for allergic rhinitis control, asthma and global.

The minimal clinically important difference was 3.25 (SD 3.77) similar to that with the German validation of 3.5^11^. This means a change in CARAT’s global score > 3.25 is the minimal difference detected by the said scale, which a patient considers as important in the control of allergic rhinitis and asthma.

The different cut-point in order to establish the control of allergic rhinitis, asthma and global were determined for the CARAT10 Spanish. The point for a controlled disease was CARAT10-rhinitis ≥ 8, sensitivity of 80% and specificity of 72%; CARAT10-asthma > 16, with sensitivity and specificity of 76%. and CARAT-global ≥18, with sensitivity and specificity of 76%.

## Supplementary information

Data Base

REPORTING SUMMARY

## Data Availability

The data that support the findings of this study are available on request from the corresponding author Diana Quijano.
