# Supplementary material for: Validation of the Spanish language version of the control of allergic rhinitis and asthma test
Source: NPJ Prim Care Respir Med. 2022 Oct 29;32:47. doi: 10.1038/s41533-022-00313-8 (PMC9617860; doi:10.1038/s41533-022-00313-8)
Supplement: Supplementary file 2 — REPORTING SUMMARY [file 41533_2022_313_MOESM2_ESM.pdf]

nature portfolio

Corresponding author(s): Diana Quijano

Last updated by author(s): Aug 15, 2022

Reporting Summary

Nature Portfolio wishes to improve the reproducibility of the work that we publish. This form provides structure for consistency and transparency in reporting. For further information on Nature Portfolio policies, see our Editorial Policies and the Editorial Policy Checklist.

Statistics

For all statistical analyses, confirm that the following items are present in the figure legend, table legend, main text, or Methods section.

n/a	Confirmed	
☐	☒	The exact sample size (n) for each experimental group/condition, given as a discrete number and unit of measurement
☐	☒	A statement on whether measurements were taken from distinct samples or whether the same sample was measured repeatedly
☐	☒	The statistical test(s) used AND whether they are one- or two-sided<br>*Only common tests should be described solely by name; describe more complex techniques in the Methods section.*
☐	☒	A description of all covariates tested
☐	☒	A description of any assumptions or corrections, such as tests of normality and adjustment for multiple comparisons
☐	☒	A full description of the statistical parameters including central tendency (e.g. means) or other basic estimates (e.g. regression coefficient) AND variation (e.g. standard deviation) or associated estimates of uncertainty (e.g. confidence intervals)
☐	☒	For null hypothesis testing, the test statistic (e.g. F, t, r) with confidence intervals, effect sizes, degrees of freedom and P value noted<br>*Give P values as exact values whenever suitable.*
☒	☐	For Bayesian analysis, information on the choice of priors and Markov chain Monte Carlo settings
☒	☐	For hierarchical and complex designs, identification of the appropriate level for tests and full reporting of outcomes
☐	☒	Estimates of effect sizes (e.g. Cohen's d, Pearson's r), indicating how they were calculated

Our web collection on statistics for biologists contains articles on many of the points above.

Software and code

Policy information about availability of computer code

Data collection	No software was used
Data analysis	file:///C:/Users/Macbook-Air/Downloads/20210522-carat%20(1).html

For manuscripts utilizing custom algorithms or software that are central to the research but not yet described in published literature, software must be made available to editors and reviewers. We strongly encourage code deposition in a community repository (e.g. GitHub). See the Nature Portfolio guidelines for submitting code & software for further information.

Data

Policy information about availability of data

All manuscripts must include a data availability statement. This statement should provide the following information, where applicable:

- Accession codes, unique identifiers, or web links for publicly available datasets
- A description of any restrictions on data availability
- For clinical datasets or third party data, please ensure that the statement adheres to our policy

I am sending in an attached file all the raw data of the patients

Human research participants

Policy information about studies involving human research participants and Sex and Gender in Research.

Reporting on sex and gender	Gender was reported in this study based on social behavior and patient-reported information.
Population characteristics	One of the variables evaluated was age.
Recruitment	The patients were recruited from the rom the authors of the Fundación Neumologica Colombiana , they were invited to participate if they had a diagnosis of bronchial asthma and allergic rhinitis as described in the study
Ethics oversight	The study was approved by the Ethics Committee of the Fundación Santa Fé de Bogotá y Fundación Neumológica Colombiana

Note that full information on the approval of the study protocol must also be provided in the manuscript.

Field-specific reporting

Please select the one below that is the best fit for your research. If you are not sure, read the appropriate sections before making your selection.

☒ Life sciences ☐ Behavioural & social sciences ☐ Ecological, evolutionary & environmental sciences

For a reference copy of the document with all sections, see nature.com/documents/nr-reporting-summary-flat.pdf

Life sciences study design

All studies must disclose on these points even when the disclosure is negative.

Sample size	A sample of ten patients was used per studied item because the scale has that number of features. The minimum sample were 100 patients who completed the required visits (14-16) and it was determined to complete the sample with 120 patients.
Data exclusions	No data were excluded
Replication	Some of the measures were replicated throughout the study.
Randomization	Does not apply
Blinding	Does not apply

Reporting for specific materials, systems and methods

We require information from authors about some types of materials, experimental systems and methods used in many studies. Here, indicate whether each material, system or method listed is relevant to your study. If you are not sure if a list item applies to your research, read the appropriate section before selecting a response.

Materials & experimental systems

n/a	Involved in the study	
☒	☐	Antibodies
☒	☐	Eukaryotic cell lines
☒	☐	Palaeontology and archaeology
☒	☐	Animals and other organisms
☐	☒	Clinical data
☒	☐	Dual use research of concern

Methods

n/a	Involved in the study	
☒	☐	ChIP-seq
☒	☐	Flow cytometry
☒	☐	MRI-based neuroimaging

Clinical data

Policy information about clinical studies

All manuscripts should comply with the ICMJE guidelines for publication of clinical research and a completed CONSORT checklist must be included with all submissions.

Clinical trial registration	The study is a validation of a scale
Study protocol	Full Trial protocol could be accessed in Spanish language

Data collection	Patient selection was done between March 2020 and January 2021, composed of 120 patients from Fundación Neumologica Colombiana
Outcomes	Based on recommendations by the Consensus-Based Standards for the Selection of Health Measurements (COSMIN), the validation of a scale was performed with a reliability evaluation, validity and sensitivity to change of the instrument as well as establishing the values for the minimal clinically important difference.
